# Children's Representations of Attachment and Positive Teacher–Child Relationships

**DOI:** 10.3389/fpsyg.2017.02270

**Published:** 2017-12-22

**Authors:** Manuela Veríssimo, Nuno Torres, Filipa Silva, Carla Fernandes, Brian E. Vaughn, António J. Santos

**Affiliations:** ^1^William James Center for Research, ISPA-Instituto Universitário de Ciências Psicológicas, Sociais e da Vida, Lisbon, Portugal; ^2^Auburn University, Auburn, United States

**Keywords:** attachment theory, teacher–child relationship, internal working models, preschoolers, verbal abilities

## Abstract

This study was designed to explore whether children's representations of attachment contribute to the co-construction of positive teacher–child relationships. An assessment of verbal intelligence was included as a predictor on the assumption that teachers might perceive themselves as having better relationships with more verbally competent children. Participants were 52 children from two pre-schools, in the district of Lisbon. The Attachment Story Completion Task (ASCT) was used to assess children's attachment security. The PCV-P (a scale developed in portuguese language) was used to describe teacher–child relationships through *teachers' ratings of child secure base behavior and emotion regulation* and the *Wechsler Preschool and Primary Scale of Intelligence* (WPPSI-R) was used to access verbal skills. Bivariate correlations showed that the teachers' rating of child secure base behavior was significantly associated with both child attachment security and verbal IQ. In a multiple regression analysis, the overall model *R*^2^ was significant, as was the interaction term showing a moderating effect of attachment security on the relation between verbal IQ and teachers' ratings of secure base. The results suggest that co-construction of a close attachment-relevant relationship with teachers in early childhood is, in part, a function of the security in the context of parent-child attachment, but also of child verbal development.

## Introduction

As attachment theory and research has expanded to consider a variety of attachment figures with overlapping, although not identical, spheres of influence (e.g., Lamb, 2005; Monteiro et al., 2010), there have been conceptual as well as empirical discussions of attachment “networks”, i.e., the sets of adults to whom a child has co-constructed an attachment (or attachment-relevant) relationship (van IJzendoorn, 2005), and the effects of secure vs. insecure relationships across all (or most) members of these networks. van IJzendoorn (2005) and others have argued that the attachment network is integrated, even if not exactly additive, and that the child's construction of an internal working model of attachment is influenced to varying degrees by all of her/his attachment relationships.

There are, nevertheless, very few studies that allow a determination of whether the contributions of each attachment relationship to internal working models is equivalent to that of all other attachments, or if they do not contribute equally, or whether there is a valid metric for weighting the influences of different attachment relationships. Regardless of how these questions are resolved, it is clear that with increasing age and maturity the number of significant relationships for a given child increases as they enter into more complex and diverse social groups beyond the original parent-child dyad(s) (e.g., Lamb, 2005; van IJzendoorn, 2005). During early childhood, the child's network of relationships will include caregivers and teachers/mentors as well as friendly relationships with peers, and by the end of adolescence most will enlarge to accommodate romantic partners (van IJzendoorn, 2005; Cyr and van IJzendoorn, 2007). At all age periods, it is likely that significant relationships will serve some attachment-relevant functions, and some may evolve to become prototypic attachment relationships, if those functions include protection, safety, and support for feelings of security.

For example, some studies examined infant and child attachments beyond the family, in family and center-based care settings (e.g., Anderson et al., 1981; Howes et al., 1990, 1998; see review by Howes, 1999) and have shown that teacher–child relationships serve very important functions for children and are recognized as attachment-relevant, even if they do not meet the strictest criteria set by attachment theory for primary attachment figures. That is to say, Bowlby (1982) stated that attachments were love relationships and that loss of an attachment figure resulted in the attached child experiencing grief and mourning responses. To the best of our knowledge there are no studies of children showing consistent preferences for comforting by a teacher vs. a parent if the child experiences distress when both are present. Neither did we find any reports of children protesting for any length of time when separated from teachers for weekends or grieving when their teacher leaves the class before the end of a term or when the child moves to a new classroom with a different teacher in sequential academic years, yet these kinds of reactions are commonplace when a young child loses a parent through abandonment, divorce, or death (Bowlby, 1982, 1989).

## Teacher–child relationships as attachment-relevant

During early childhood, children are still developing their capacities for autonomous functioning and self-regulation (Sierra, 2012). By acting as a secure base for exploration, allowing sufficient independence to discover and manipulate materials and play contexts, while at the same time monitoring and scaffolding activities within these contexts, caregivers/teachers support growth of the child's cognitive and social competencies (Verschueren and Koomen, 2012). Research has shown that the same interaction qualities (e.g., sensitivity to communicative signals coupled with prompt and effective responses contingent on those signals) underlying the co-construction of secure attachments in the family also facilitate the co-construction of teacher–child relationships (e.g., Goossens and van IJzendoorn, 1990). However, and different from the earliest attachments, a child's secure base relationship with a teacher is inevitably shared with peers who are also resident in the classroom (Lamb, 2005) and the degree to which the teacher is shared increases dramatically over the years of schooling, as curriculum requirements shift from more autonomous exploration to didactic instruction and as the number of teachers with whom a child interacts on a daily basis increases (Verschueren and Koomen, 2012).

For these reasons, some researchers (e.g., Riley, 2011; Sierra, 2012; Verschueren and Koomen, 2012) consider teachers to be attachment- relevant (or “secondary” attachment figures to use Bowlby's term). But others point out that children whose attachment with the primary figure (mothers in most studies) is secure also tend to co-construct a secure relationship with their teachers during early childhood (e.g., DeMulder et al., 2000; Chung et al., 2005; Rydell et al., 2005), which may indicate that the child is actively reproducing the earlier existing primary attachment organization with a new adult. And still others fail to find concordance with regard to security between mothers and teachers (e.g., Cassibba et al., 2000). In a longitudinal study, O'Connor and McCartney (2006) showed that insecure attachment significantly predicted the quality of teacher relationship at 54 months, kindergarten, and 1st grade. Also, Buyse et al. (2011) showed that closeness between child and teacher may compensate more problematic mother child relations.

Regardless of how the question of the status of teacher–child relationships as attachments is resolved, there is no argument concerning the importance of those relationships for grounding the child's experience of educational contexts in early and middle childhood and the attachment framework provides some interesting tools for exploring the impact of teacher–child relationships. Thus, the central question we explore in this study concerns the construction of a teacher–child relationship resembling a secure base/attachment relationship with primary caregivers and the child attributes predictive of this relationship. We anticipated that the children's representations of attachment would contribute to the co-construction of positive teacher–child relationships, as characterized by the teachers themselves.

## Teacher–child relationships' quality and children's verbal abilities

School environments are especially interesting contexts in which attachment-relevant relationships may be co-constructed because children spend up to seven hours per day in educational settings from early childhood through late adolescence (at least in western developed countries). Moreover, school contexts pose many challenges that constrain the trajectories of children's emotional development (Verschueren et al., 2012). Over the last 30 years, with the increase of maternal employment rates, children started attending (pre)school at increasingly earlier ages (Lamb et al., 1990). Preschool care and early education has become increasingly specialized, as working parents have less and less time to serve as primary caregivers and educators for their children. With this temporal change in the modal forms of early childcare and education, research concerning teacher–child relationships has become a central topic in the developmental and education sciences. Research has highlighted the importance of teacher-student relationship quality in all school age groups (Cyr and van IJzendoorn, 2007; Sabol and Pianta, 2012). In fact, the quality of this relationship, has been identified as a critical support for academic success (Martin et al., 2007), school adaptation (Baker et al., 2008), good classroom management (Riley, 2011), and better peer relationships (Verschueren et al., 2012).

However, several studies found that the teacher student relation is related to both contextual and individual characteristics of the child (Hamre and Pianta, 2006). For example, the time children spend at school, motivation to learn or disruptive behavior can influence teacher behavior and consequently teacher–child relation. School teachers are obligated to emphasize the educational goals of the larger community and spend less and less time enacting the protective and nurturing roles characteristic of attachment figures. So, another aim of the present study is to verify the assumption that teachers would perceive themselves as having a better quality relationship with children who display ability to achieve the curriculum goals more rapidly than their peers.

Additionally, poor language skills, and in particular poor verbal comprehension at school entry are an indicator of low school readiness and a risk for subsequent academic problems (NICHD Early Child Care Research Network, 1997). Partenio and Taylor (1985) found that IQ was the best predictor of teacher ratings on classroom performance, motivation to learn, and learning potential of students. On the other hand Spilt et al. (2015) reported that there were reciprocal associations between close teacher–child relationships and language development in students. Hence, children's verbal abilities seem to be an important variable to include in models of teacher–child relationships.

## Methods

### Participants

The participants were 52 children (25 boys) from two preschools in the district of Lisbon. All children were of caucasian ethnicity and were between 4 and 5 years old (*M* = 4.4, *SD* = 0.20). The age of entry into daycare ranged from 6 to 30 months (*M* = 8.67, *SD* = 6.59) and children spent between 4 and 10 h (*M* = 7.59, *SD* = 1.62) in non-parental care each weekday. Participants are part of a larger longitudinal study of child social and emotional adaptation in the family and the peer group. Children were from two-parent families: mothers were between 26 and 48 years (*M* = 34.95, *SD* = 4.33) and fathers between 28 and 63 years (*M* = 37.48, *SD* = 6.08). The educational backgrounds of mothers varied between 7 and 23 years of education (*M* = 15.46, *SD* = 3.34), as well as for the father (*M* = 14.77, *SD* = 3.17). All families were middle class by the standards of the local community. Four teachers also participated in the study. All of these had completed a University Degree in preschool Education. The age of the teachers ranged between 29 and 50 years of age and service time between 6 and 25 years.

### Procedures

The research team contacted the preschool centers using a public list available in official registries and invited them to participate in the survey. After ethical approval from the Comité de Ética do Centro de Investigação do ISPA and signed informed consent by the parents, children's assent was obtained before the individual interviews. The two instruments were applied individually to the child by independent research assistants with specific training: first the Attachment Story Completion Task (ASCT) followed by the Wechsler Preschool and Primary Scale of Intelligence (WPPSI-R). Both the WPPSI and the ASCT administration were made in a room of the childcare centers and data were collected during the spring term. The teacher completed a questionnaire for each child at the end of the spring semester.

### Measures

Attachment Story Completion Task (ASCT, Bretherton et al., 1990). The ASCT was used to assess children's symbolic attachment representations. A series of story-stems were presented to the child to elicit narratives regarding attachment behaviors toward caregivers. Story stems were presented using dolls and household props, including a mother, father, child, sibling, a pet dog, kitchen equipment, living room and bedroom furniture, etc. The child doll was the same sex and ethnicity as the child being assessed. The assessments took place in a quiet area outside the classroom or in the classroom at a time when other children were elsewhere. The interviewer invited the child to play the story completion game together, with the interviewer beginning each story and the child finishing the story. The child was first presented with a story stem about a birthday party with a pleasant but non-attachment related theme. This was intended as a warm-up story and was not scored. The child was then presented with the five primary attachment-related story-stems (e.g., parents leave for an overnight trip while the child and a sibling stay at home with an aunt) and asked “show me and tell me what happens next.” Non-directive questions such as “Does anything else happen in the story?” or “What are they doing?” were used to facilitate the child's narrative production. The story was completed when the child indicated that he/she was finished. All stories were rated independently by two trained coders who were blind to any other information about the child, Inter-observer reliability was assessed through Intra Class Correlations, and all 5 narratives showed strong significant coefficients, ranging from 0.78 to 0.93. Stories were rated from videotapes on an eight-point scale for *Security*, developed by Maia et al. (2009). This scale was inspired by Heller's (2000) work who, based on preexistent contributions (Robinson et al., 1992; Page and Bretherton, 1993; Golby et al., 1995), proposed a quite comprehensive coding system which included identify cation of general and summary themes (e.g., prosocial, obedience/discipline, aggression, danger etc.), a broader assessment of narrative aspects (e.g., parental representations, type of story resolution) and performative relevant elements (e.g., overall emotional expressiveness, emotional knowledge, interaction with the interviewer, non-verbal behavior, investment in performance, fluency and avoidance), together with coherence and security scales. The *Security* score is a broad parameter which considers plot coherence and the extension to which each attachment-related challenge is acknowledged and successfully dealt by the child, derived after a global evaluation of the narrative and of the performance at the task is done. Comparing a sub-set of preschoolers' ASCT stories from middle-class and from disadvantaged Head-Start attendants', Heller (2000) reported that security scores were significantly associated to maternal sensitivity/elaborative style during mother–child talks about past events and to the quality of the mother–child narrative co-construction around a separation–reunion theme. On their turn, secure and coherent attachment story resolutions were predicted by precedent observational measures of child–mother attachment. In sum, the *Security* score is a broad dimension that considers how effectively the child addressed the major issues in the story and uses the caregivers as secure base.

*Teachers' ratings of child secure base behavior and emotion regulation* (PCV-P). The PCV-P (Dias et al., 2004) was developed in Portuguese language (original title of the scale is “*Percepção do comportamento de vinculação*”) and the scale is based on items from the attachment Q-sort (AQS; Waters et al., 1991; Waters, 1995). A factor analysis identified two dimensions describing the child teacher relationship: (1) use of the teacher as a Secure Base (SB), consisting of 16 items which measures exploration behavior and the use of the teacher as a secure base (e.g., when he is disturbed he accepts my comfort), an d (2) child Self-regulation of Emotion (ER), consisting of 12 items which seek out to evaluate emotion regulation in relation to the child's relationship with the teacher and with their peers (e.g., the student tries to solve problems with aggressive peers with a nonviolent approach). The 28 items of the scale are assessed by teachers according to a 5-point scale from 1 = extremely uncharacteristic of this student, 5 = extremely characteristic of this student. The teachers were instructed to rate the child's behaviors toward herself and to his/her peers, as exhibited and observed by the teacher in recent months.

They were told that the questionnaire items were intended to characterize the relationship they had established with each participating child in the classroom and their responses should reflect each child's behavior over the past 2 months of the term. In the developmental study (Dias et al., 2002), this measure showed good internal consistency for both the Secure Base and the Emotion Regulation subscales, with Cronbach's alpha coefficients of 0.93 and 0.89, in the first study and 0.86 and 0.84, in a second study, respectively. In the present study, Cronbach's alpha coefficients for both subscales were also satisfactory (0.84 for the SB dimension and 0.78 for the ER dimension).

*Wechsler Preschool and Primary Scale of Intelligence:* WPPSI-R (Seabra-Santos et al., 2003). The Portuguese version of the WPPSI was used to control for the possible effect of individual differences in linguistic and verbal skills on the child Security and teacher SB and ER scales, results were similar to the ones reported in the Portuguese adaptation (*M* = 102.1, *SD* = 13.09). The WPPSI consists of 12 sub-tests of which 6 subtests are perceptual-motor and 6 subtests are verbal. The scores of the 12 subtests are combined in three composite scores: General Intelligence Quotient (IQ), verbal IQ and perceptual-motor IQ. In this study only the verbal IQ was used. The verbal IQ score is composed by the following subtests: information, vocabulary, word reasoning, comprehension, similarities and picture naming.

## Results

Preliminary analyses tested for relations between the demographic indicators (i.e., mother and father age, teachers' years of experience, number of months the child had been enrolled in daycare prior to assessment, number of hours in day care) and teachers' ratings of child secure base behavior and emotion regulation and attachment representations. In no instance were the teachers' ratings or the attachment representations associated significantly with these demographic variables.

### Associations among the study variables

As shown in Table 1, the teacher-rated secure base is significantly associated with both attachment security and verbal IQ. Attachment security is also associated with verbal IQ. Finally, emotional regulation is associated with gender: the teachers characterized girls as being better able to regulate emotion than boys.

**Table 1:** Correlations between all variables in the study.

**Variables**	**1.**	**2.**	**3.**	**4.**	**5.**
1. Attachment Narrative security	–	0.35^*^	0.17	0.37^*^	−0.06
2. Secure base		–	0.42^**^	0.46^**^	−0.06
3. Emotional Regulation			–	−0.18	0.17
4. Verbal IQ					−0.08
5. Gender (0 = boys; 1 girls)					–

* p < 0.05;

** *p < 0.01*.

### Predicting teachers' ratings of secure base behavior

Table 1 shows that the child's representation of a secure attachment is significantly correlated with both teachers' ratings of secure base behavior and child's verbal IQ. Consequently, in the next analysis we tested whether or not secure attachment representations interacted with verbal IQ to predict teachers' ratings of child's secure base behavior. To test this possibility, we regressed the teachers' ratings of child's secure base behavior on the ASCT security score, the verbal IQ score, and their interaction. A regression model that included an interaction term for attachment security representations × verbal IQ was calculated (Table 2). In this analysis, both main effects and their interaction were significant predictors of the teachers' ratings. As shown in Table 2, both main effects and their interaction were unique, significant predictors of the teachers' ratings of secure base behavior in the regression analysis, suggesting a possible moderating effect of attachment security on the association between verbal IQ and ratings of child secure base behavior.

**Table 2:** Model predicting Teacher Characterization of Child Secure Base Behavior with Interaction term.

	**Dependent Variable**	
	**Secure Base**	
	**Beta**	***t***
Attachment Narrative security	0.27	2.0^*^
Verbal IQ	0.38	2.8^**^
Attachment Narrative security X IQ	−0.26	2.1^*^
Model *R*^2^	0.31^***^	

* p < 0.05;

** p < 0.01;

*** *p < 0.001*.

**Figure 1:**
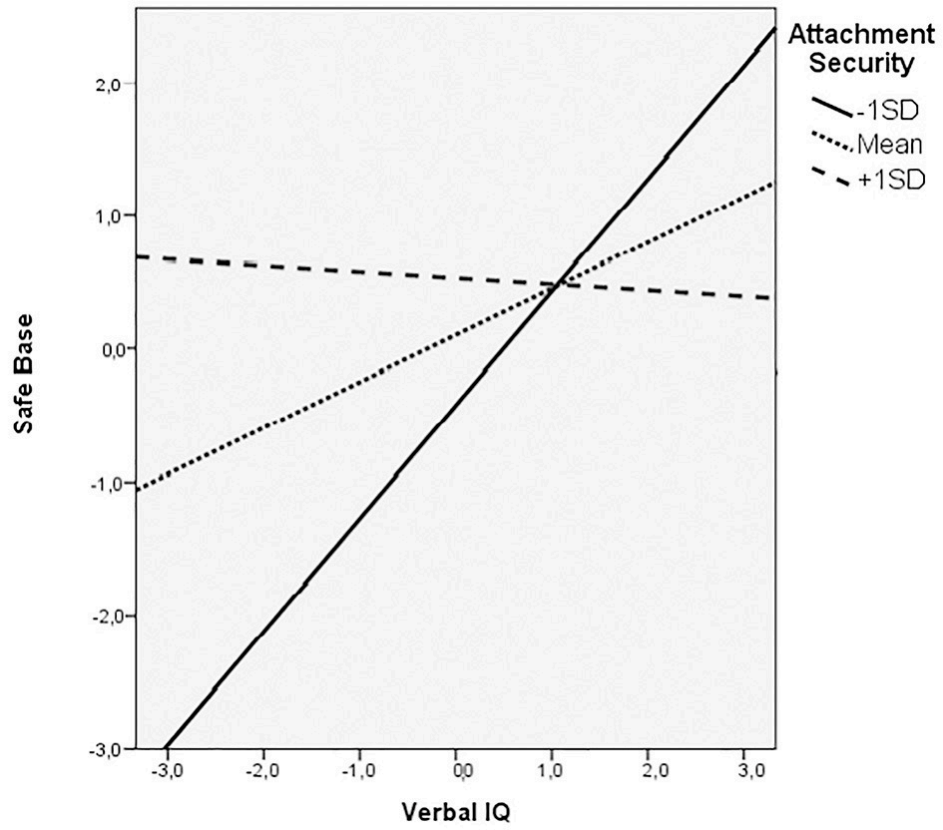
Plot of slopes of Relations between Teacher–Child Relationships and Verbal IQ for Three Levels of Attachment Security.

To test for moderation effects, the relation between verbal IQ and teachers' ratings of secure base behavior was examined at three levels of attachment security. We plotted the simple slopes between teachers' ratings of secure base behavior and verbal IQ at three different levels of security for child's representations of attachment (Figure 1). Because all the variables are standardized with average of 0 and Standard deviation of 1, the values of attachment security plotted are −1 SD (low), 0 (middle) and +1 SD (high). The estimates for the relation between verbal IQ and teachers' ratings of secure base behavior at the low, average and high values of attachment security were: *beta* = 0.70 (*p* < 0.05), *beta* = 0.36 (*p* < 0.05) and *beta* = −0.11 (n.s.), respectively. That is to say, the relation between verbal IQ and teachers' ratings of secure base behavior was greatest when attachment security is low, and remains significant when attachment security is at the intermediate level. However, when the child's representation of attachment security was high (over 1 SD above the mean for the sample) the relation between verbal IQ and teachers' ratings of secure base behavior was not significant. *Post-hoc* tests contrasting the significance of the differences between the estimates at the three levels revealed that only the difference of estimates between high and low child representations of attachment security was significant (*z* = 1.88, *p* < 0.05).

In addition, an examination of the scatterplot for the verbal IQ × Teacher characterization of the child's secure base behavior, showed that children with low scores (i.e., <−1 SD) for both attachment security representations *and* verbal IQ had lower scores (on average) for teacher rated secure base behavior than children with higher scores for attachment security representations (i.e., mid-range and high). Moreover, *all* children with high attachment representation scores (i.e., >1 SD) had teacher rated secure base scores above the group mean, although they were heterogeneous with respect to verbal IQ. This is the most likely reason why the sign on the interaction term is negative.

## Discussion

In the present study, we aimed to assess the relation between quality of children's internal representations of attachment (i.e., security) and the quality of teacher–child relationships, because we know that the quality of these relationships has a considerable impact on academic successes, as well as cognitive, affective and emotional growth and also influence children's social adjustment in school, especially in the primary school years (Hamre and Pianta, 2006; Martin et al., 2007). We found non-significant relations between adult demographic factors, such as age of the teacher and the teacher's characterization of the child's secure base behavior with her. Teachers did tend to describe girls as better able to regulate emotions then boys. Nonetheless, non-significant sex differences were found for the teachers' ratings of children using her as a secure base for exploration.

As in previous studies we found significant association between teacher's ratings of child's secure base behavior and the quality of child's attachment representations from the ASCT (e.g., Sroufe, 1983; Howes and Hamilton, 1992; DeMulder et al., 2000; Rydell et al., 2005; Berlin et al., 2008; Sierra, 2012). However, we also found significant correlations between verbal IQ and both children's representations of attachment and teachers' ratings of the child's secure base behavior. It may be that teachers are sensitive to children's verbal skills and tend to see children who are achieving academic goals on or ahead of schedule as children with whom they have positive relationships. That is, children with high verbal capacity were also characterized by their teacher as better able to use her as a secure base for exploration, independent from their attachment history. Thus, when the child articulates a secure representation of attachment (i.e., high scores when responding to the ASCT), teachers tend to characterize him/her as using her (the teacher) as a secure base, independently of verbal ability. However, when the child fails to articulate a secure attachment representation (i.e., mid-range and lower ASCT scores) but *does* demonstrate a high level of verbal ability, teachers also tend to describe the child as being able to use them as a secure base. These findings suggest that secure attachment representations are one important pathway to co-constructing a positive and close relationship with teachers, but also that teachers tend to see themselves as having positive, attachment-like relationships with children having greater verbal ability.

Overall, our data suggest that co-construction of a close, attachment-relevant relationship with a teacher in early childhood is, in part, a function of the quality of parent-child attachment relationships, as indexed in this study by the quality of attachment representations elicited by the ASCT story stems. However, child attachment representations are not the sole route to a teacher–child relationship that teachers characterize as being attachment-relevant (i.e., the child organizes secure base behavior with reference to the teacher). It could be that more verbally able children initiate contact with their teachers to inquire about classroom activities, play opportunities, or to adjudicate disputes more frequently than do less verbally capable children. Alternatively, teachers may tend to prefer more verbally intelligent children and feel that the relationship between them is positive and warm, without regard to the frequency of interactions between them. And, as noted above, when attachment representations do not suggest a history of secure attachments and children demonstrate lower verbal intelligence, teachers are less likely to characterize their behavior as indicating a secure base relationship with the teacher. These possibilities should be considered as research on teacher–child relationships goes forward. Whatever the reason, it remains the case that preschool and elementary school teachers support both children and their families when they nurture and protect young children and that the relationships formed in the early preschool and elementary school years can support or undermine future cognitive, social, and affective development of the child (Berlin et al., 2008).

We also recognize the there are limitations and constraints on the generality of our findings. For example, we only have teachers' self-reports for teacher–child relationship quality. It will be important in future studies to include assessments of teachers' sensitivity and direct observations of the children's behavior with reference to the teacher. In addition, the children recruited to this study came from predominantly middle class families who were attending private, non-profit early childhood education programs. It is possible that children from less advantaged families might have lower average verbal intelligence scores than children in this study and, if so, the quality of their teacher–child relationships could be more strongly associated with the security of attachment representations than with verbal intelligence. Finally, all measures used in this study were obtained concurrently; longitudinal studies in which attachment security and verbal intelligence are assessed on multiple occasions, ideally starting prior to entry into childcare, will be important for disentangling the implications of attachment security and verbal intelligence for teacher–child relationship quality.

There is an ample evidence base to assert that the quality of teacher–child relationships in both preschool and elementary school settings is a critical influence on the child's adaptation to and success in those settings. Because children enter school settings from diverse backgrounds and with diverse sets of experiences grounding their social and cognitive skills, it is important that teachers are sensitive to this diversity and are prepared to meet the developmental needs of every child in their classrooms. This study highlights two domains of child competence, security of attachment representations and verbal intelligence that are associated with teachers' characterizations of children treating them as a secure base. These findings will be useful when designing curricula for training early childhood educators to be more aware of the meanings of children's behavior as well as the possibility that they may be more drawn to children demonstrating greater verbal ability. Our findings may also be useful for planning intervention programs for children who are having difficulties adapting to the preschool and/or primary school contexts.

## Ethics statements

This study was carried out in accordance with the recommendations of Comité de Ética do Centro de Investigação do ISPA with written in review informed consent from all subjects. All families gave written informed consent in accordance with the Declaration of Helsinki.

## Author contributions

MV: Overview of data collection, writing of the manuscript, review of the manuscript; NT: Statistical analysis, help in writing, review of the manuscript; FS: Data collection; CF: Data collection; BV: Writing of the manuscript, review of the manuscript; AS: Overview of data collection, writing of the manuscript, review of the statistical analysis, review of the manuscript.

### Conflict of interest statement

The authors declare that the research was conducted in the absence of any commercial or financial relationships that could be construed as a potential conflict of interest.
